# Asymmetry of attentional set in rhesus monkeys learning colour and shape discriminations

**DOI:** 10.1080/17470210600971485

**Published:** 2006-12-22

**Authors:** Mark G. Baxter, David Gaffan

**Affiliations:** Oxford University, Oxford, UK

## Abstract

We trained rhesus monkeys on six visual discrimination problems using stimuli that varied in both shape and colour. For one group of animals shape was always relevant in these six problems, and colour always irrelevant, and for the other animals vice versa. During these “intradimensional shifts” (ID) the problems were learned at equal rates by the two groups, shape-relevant and colour-relevant. We then trained three further problems in which the other dimension was now relevant (“extradimensional shifts”, ED). The animals showed slower learning when shifting from colour-relevant to shape-relevant, but not when shifting from shape-relevant to colour-relevant. These results show that monkeys' ability to selectively attend to a relevant stimulus dimension and to ignore an irrelevant dimension depends on the experimenter's choice of relevant and irrelevant dimensions.

Evidence that animals can learn to attend to a stimulus dimension comes from experiments employing an intradimensional/extradimensional shift paradigm (Mackintosh, 1974). In one version of this paradigm the subjects initially learn discrimination problems (“intradimensional shifts”, ID) in which the stimuli vary in at least two dimensions, one of which is always relevant to problem solution and the other of which is always irrelevant to problem solution (George & Pearce, 1999). Subsequently the animals learn a new problem with stimuli varying in the same two dimensions, but with the relevant and irrelevant dimensions now changed, the previously relevant dimension becoming irrelevant and the previously irrelevant dimension becoming relevant. Slower learning in this “extra-dimensional shift” problem (ED) shows that, during ID learning, the subjects learn to attend selectively to the then relevant dimension and thus to ignore the then irrelevant dimension.

However, this pattern of results is not obtained in all such experiments. Rats can show the opposite pattern, a superiority of ED over ID, in spatial learning (Trobalon, Miguelez, McLaren, & Mackintosh, 2003). Similarly, studies of human attentional processes show that one should not expect all pairs of stimulus dimensions to produce the same pattern of results when their relevance is varied in this way. For example, Garner (1974, pp. 126–127) describes an experiment in which human observers classified single coloured patches according to either their brightness (“value”) or their saturation (“chroma”). Classification speed was reduced in the case where both dimensions varied but only one was relevant to classification, by comparison to the case where only the relevant dimension varied. This indicates that, in Garner's terms, these two dimensions are integral; that is, the subject cannot attend to just one of them. In a survey of results from a wide range of auditory, visual, and linguistic dimensions of variation using several different dependent measures, Garner (1974) concluded that the difficulty of attending selectively to one stimulus dimension while ignoring another varied widely according to what pair of stimulus dimensions was used. Furthermore, in many such pairs of dimensions the attentional separability of two dimensions is asymmetrical: For example, in auditory discrimination human observers could ignore the consonant of a spoken syllable while discriminating its pitch, but not vice versa. Garner predicted (1974, p. 137) that shape and colour should behave in this way—that is, it should be possible to discriminate colour while ignoring shape, but not vice versa. In order to test Garner's prediction in the rhesus monkey, we used an ED/ID paradigm in which the two stimulus dimensions were colour and shape.

## Method

### Subjects

A total of 8 male rhesus monkeys (*Macaca mulatta*), 3.0–4.1 kg (23.5–25 months old) at the beginning of behavioural training, participated in this study. The monkeys were housed socially in a single troop, in an indoor enclosure attached to standard caging. Water was available ad libitum in the home enclosure; each monkey's daily food ration was delivered in the test box and was supplemented with fruit and forage mix in the home enclosure. A 9th monkey in this group began the experiment but was excluded because he did not show reliable discrimination performance.

### Apparatus

Behavioural testing took place in an automated apparatus. Each monkey was taken from the home enclosure into the test cubicle in a wheeled transport cage, which was fixed in front of a video-display unit with a touch-sensitive screen (380 × 280 mm, 800 × 600-pixel resolution). The monkey could reach through horizontally oriented bars (approximately 45 mm apart) at the front of the cage to reach the screen and the rewards. Stimulus presentation, recording of touches to the screen, and reward delivery were all under computer control. A pellet dispenser delivered 190 mg banana-flavoured or sugar pellets (P. J. Noyes, Lancaster, NH) into a food cup located below the touchscreen. Pellet delivery produced a click from the pellet dispenser as well as a 500-ms tone from the computer. A metal “lunchbox” (approximately 200 × 100 × 100 mm) was located to the left of the food cup and was filled with a mixture of wet monkey chow, seeds, apple, banana, orange, nuts, and dates. Infrared cameras positioned at different locations within the test cubicle permitted observation of the monkey while it was performing the task. The entire apparatus was located in an experimental cubicle that was dark except for the illumination of the video screen.

### Behavioural testing

#### Pretraining

After monkeys were shaped to enter the transport cage from their home enclosure and were reliably taking food in the test cubicle, pretraining began. First, reward pellets were delivered on a variable-interval (2-min) schedule to accustom them to take pellets in the test box. After several days of pellet training, the touch-screen was activated, and the screen was filled with an array of different-coloured alphanumeric characters on a black background (in a different size and typeface from those used in the main task). Touches to any location on the screen resulted in pellet delivery. In the third and final stage, single clip art images (128 × 128 pixels) were presented in random locations on a grey background. When the monkey touched the image, a pellet was delivered, and a new clip art image was displayed after a 5–10-s intertrial interval (ITI). If the stimulus was not touched within 180 s, it disappeared, and a new one was presented after the ITI. Touches to the screen during the ITI reset the ITI countdown. Once each monkey was reliably completing 50 trials in a session in this final pretraining task, training on the main task began.

#### Colour and shape stimuli

Stimuli were produced in Adobe Photoshop. Alphanumeric characters in 144-point Arial font were centred within a 128 × 128 pixel grey square, the same colour as that used as the screen background during the final pre-training task and the main discrimination task. A grey background was chosen so that both dark and light colour shades could be used. A total of 20 different alphanumeric characters (1, 2, 3, 4, 5, 7, 8, A, F, H, K, P, Q, R, W, X, #, $, %, +) served as shapes, and 20 colours (including black and white) were chosen that were discriminable from each other on the video screens used for behavioural testing as judged by two human observer. For each monkey, a random sequence of colours and shapes was generated, unique to that monkey, so that each colour and shape was used once, and these stimuli were assigned to pairs for discrimination problems (so a total of 10 pairs were possible, although only 9 were used in this task).

#### Discrimination learning

For each problem, the next two colours and two shapes were taken from the sequence generated for each monkey. Two stimuli appeared on the screen on each trial, one on the left side and one on the right; stimuli were centred top to bottom, and the centre of each stimulus was 200 pixels from the centre of the screen. Either shape could appear in either colour, and the assignment of the two shapes to the two colours was varied across trials in a pseudorandom sequence such that each possible pairing occurred equally often in each session. One of the two shapes or colours was randomly designated as correct for each problem for each monkey. The relevant dimension for a particular discrimination problem was either colour or shape. As an example, one problem could consist of the shapes “A” and “1” and the colours red and orange. On a particular trial, the monkey could see a red A and an orange 1, or an orange A and a red 1. If shape was the relevant dimension for that monkey, the A (for example) would be correct regardless of whether it was red or orange. Alternatively, if colour was the relevant dimension for that monkey, the red stimulus (for example) would be correct regardless of whether it was an A or a 1.

A total of 50 trials were given in each test session. Each trial lasted until the monkey touched one of the two stimuli. If the monkey chose the correct stimulus, a pellet was delivered, the incorrect stimulus disappeared, and the correct stimulus remained on the screen for 1 s, then the correct stimulus disappeared, and a 10-s ITI began. If the monkey chose the incorrect stimulus, both stimuli disappeared, no pellet was delivered, and a 20-s ITI began. On the last trial, after the final pellet was delivered, the lunchbox opened, and the screen turned black. If the monkey made an error on the 50th trial, additional trials were given until a correct response was made, then the session terminated with lunchbox delivery as before. Two monkeys developed side biases during training on their first problem, so a correction procedure was initated for several test sessions such that trials performed incorrectly were repeated exactly until a correct response was given. Errors made in these correction trials were not included in errors to criterion (see Results). Training continued on each problem for a minimum of 2 sessions and until a criterion of 90% correct responses was achieved in one test session. One test session was given daily, 5–7 days a week. Because monkeys were moving freely in the transport box in front of the touchscreen, could take as long as they wanted to respond, and were free to respond with either hand, response latency data were not collected.

Monkeys first encountered a series of six discrimination problems in which colour or shape was the relevant dimension. For 5 monkeys, colour was the relevant dimension in the first six problems; for the other 3 monkeys, shape was the relevant dimension in these problems. Three further discrimination problems were then presented in which the relevant dimension switched for each monkey (from colour to shape, or vice versa). Errors to criterion, including errors committed in the criterion session, were analysed as the dependent measure of discrimination learning rate and were log-transformed to adjust for increases in variance proportional to increases in the size of means.

## Results

Monkeys improved their performance across the six initial colour or shape discriminations. This may reflect acquisition of discrimination learning set, formation of an attentional set to the relevant colour or shape dimension, or both. Repeated measures analysis of variance (ANOVA) of the six initial problems revealed a main effect of problem, *F*(5, 30) = 5.74, *p* = .001, but no effect of colour/shape dimension or interaction of these factors (*F*s < 1).

**Figure 1:**
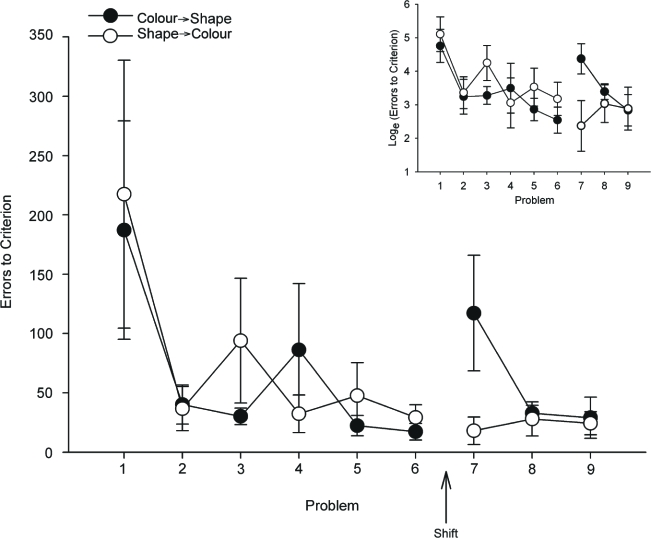
Errors to criterion for 5 monkeys that shifted from colour to shape problems (closed symbols) and 3 monkeys that shifted from shape to colour problems (open symbols). The shift took place after the sixth problem was learned. The inset graph shows log_e_-transformed errors.

Performance on the first problem after the change in relevant dimensions differed depending on the direction of the dimensional switch. Performance of shape discriminations was difficult for monkeys that had been doing colour discriminations, but monkeys that had been performing shape discriminations found colour discriminations easy. Repeated measures ANOVA revealed a trend towards a main effect of colour/shape for the three problems learned after the dimension switch, *F*(1, 6) = 5.37, *p* = .06, but no main effect of session (*F* < 1) or interaction of these factors, *F*(2, 12) = 1.94, *p* = .19. However, direct comparison of the last problem learned before the shift with the first problem learned after it revealed a significant interaction of shift direction (colour to shape vs. shape to colour) and problem, *F*(1, 6) = 6.65, *p* = .042. This difference was attributable to increased difficulty of shape discrimination for monkeys that had been discriminating colour, paired *t*(4) = 2.93, *p* = .043; monkeys in the converse condition did not find a colour discrimination any more difficult than their last shape discrimination, paired *t*(2) = 0.99, *p* = .43. Raw and transformed errors to criterion across the nine problems are plotted in Figure 1.

## Discussion

Using an ED/ID shifting design, rhesus monkeys apparently show an asymmetry in attentional set formation to colour versus shape stimuli, at least with the particular stimuli that we used. This is not obviously due to differences in the intrinsic associability or salience of the stimuli. Although arguably colour might have been easier to discriminate or more salient than shape, which would explain the asymmetry in the extradimensional shift, no statistically significant differences were noted in the acquisition of the problems. Such effects may only be apparent on the first problem encountered of each kind, but the numerical difference between colour and shape discrimination on the first problem encountered (means of 187 vs. 217 errors to criterion for colour and shape, respectively) is dwarfed by the difference between colour and shape problems after the shift (means of 18 vs. 117). Thus, it is difficult to conclude that a large difference in the extradimensional shift effect is the result of a subtle difference in acquisition rate of colour vs. shape problems. In any case, the detection and exclusion of such effects is probably impossible in designs of this type that use small numbers of monkeys.

It is possible that the similar acquisition rates of colour versus shape problems are due to different factors. For example, an attentional set to the dimension of colour might have been formed, resulting in an impairment in shifting to the previously irrelevant dimension (shape), whereas improved performance across shape problems may be predominantly a function of discrimination learning set rather than selective attention. Alternatively, and more parsimoniously, one may hypothesize that perceptual interactions between colour and shape stimuli, at least with the particular examples we used, are intrinsically asymmetrical.

Experiments on human attention have shown that, when a set of stimuli is created by combining the values of two stimulus dimensions, the stimulus dimensions may interact with each other in several, qualitatively different, possible ways (Garner, 1974). For example, the dimensions may be separable, meaning that each dimension can be attended to while ignoring the other, or integral, meaning that this is not possible. On the basis of Garner's findings one should not expect extradimensional shifts in discrimination learning always to be more costly than intradimensional shifts, as that pattern can only be seen with separable dimensions. Our present results indicate what Garner (1978) called an “asymmetrical integral” interaction, in which one dimension can be ignored while processing the other, but not the other while processing the one (Garner, 1978, p. 289).

This hypothesis has a few implications for the behavioural analysis of ED/ID shift learning and the use of these types of tasks to probe the function of neural systems. We would argue that the concept of “attention to perceptual dimension” is not entirely secure if its presence depends on the careful choice of stimulus configurations. Based on the human psychophysical literature (Garner, 1974, 1978) and our results, any combination of physically separate stimuli can produce any kind of interaction between them. Thus, there does not seem to be any reason to isolate the case in which these interactions are symmetrical and where prior learning about one aspect of a stimulus retards learning about a different aspect of that stimulus (as in the standard ED/ID design) as indicating a psychological process of attention to perceptual dimensions of stimuli.

In the Wisconsin card sorting test (WCST), humans sort stimulus displays according to the colour, shape, or number of identical coloured shapes (Milner, 1963). Because the dimensions we used, colour and shape, are two of the dimensions used in the WCST, our results demonstrate that it is unsafe to assume that brain lesion effects in the WCST arise from a process, general to all stimulus dimensions, of dimensional attention. Roberts and colleagues (Roberts et al., 1994; Roberts, Robbins, & Everitt, 1988) have suggested that an ED/ID shift task in marmosets is analogous to the WCST in human subjects and can therefore be used to study in monkeys the neural basis of performance in the WCST task. However, we have seen that there are severe limitations on the generality of the phenomenon of attentional set towards a relevant stimulus dimension. It therefore seems unsafe to assume that humans performing the WCST are necessarily forming an attentional set. They could sort according to a relevant stimulus dimension while still attending to an irrelevant stimulus dimension. Presumably this is the way our monkeys learned our discrimination problems with shape relevant: For example, they might learn that a green A and a red A are both rewarded and that a green B and a red B are both unrewarded, rather than learning that A is rewarded and that colour is irrelevant. We hasten to add that it has been shown that marmosets do not use this strategy to perform the ED/ID task (Roberts et al., 1988), because their discrimination performance is unaffected by a change in the irrelevant stimulus dimension; however, the stimuli used in that task are composed of separate, superimposed lines and shapes, rather than coloured shapes. A more secure way to study the possible neural bases in monkeys of performance analogous to the WCST is to train rhesus monkeys on a task more directly modelled on the WCST itself (Mansouri & Tanaka, 2002) in which working memory for rule is required.

Other features of our design differed from the common ED/ID task and merit comment. Normally, this task is given in a within-subjects fashion, where the relevant dimension is introduced in a simple discrimination (SD) phase, followed by the introduction of the irrelevant dimension, followed by one or more ID problems and reversals of those problems, and finally an ED shift. This design was developed to maximize the amount of behavioural data that can be collected in a single subject, which of course is a paramount concern in studies using experimental animals or humans with various kinds of brain damage or neuropsychiatric conditions, which may be rare. We did not include reversals of discriminations during the ID phase, because we were concerned that in future studies that might use this paradigm with monkeys with various cortical lesions that impairment in reversal learning would increase the time they spend exposed to the dimension that is relevant in that phase. Indeed, it has been proposed that exposure to reversals may be necessary for forming an attentional set (Colacicco, Welzl, Lipp, & Wurbel, 2002), although it is unclear why, in our experiment, reversals would differentially affect set formation to colour versus shape. Similarly, we did not include a separate SD phase or preliminary training on discrimination learning before beginning this task—the monkeys were behaviourally naive and had only been trained to respond to coloured symbols or clip art shapes on the touchscreen. Because in the common ED/ID design an SD phase is provided to orient the animals to the relevant stimulus dimension before irrelevant elements are introduced, this means that they have had more exposure to that dimension before the EDS is presented; thus, ED shift effects could be a consequence of reduced exposure to the irrelevant stimulus dimension (A. Duffaud & D. George, personal communication, April 13, 2006). Finally, it has been argued that ED/ID differences in within-subject designs could result from proactive interference across training and that between-subject comparisons with a final ID shift must be provided to exclude this possibility (Brigman, Bussey, Saksida, & Rothblat, 2005). This is also a legitimate criticism of our design, although we cannot see why such effects would depend on which dimension was relevant during the acquisition of ID discrimination problems, nor was there any apparent increase in difficulty across ID problems, as would be expected if there were a buildup of proactive interference. Taken together, we suggest that the interpretation of ED/ID differences in the paradigm as it is commonly presented may not always be completely straightforward, even if one chooses dimensions that are equally difficult to learn about and that produce symmetrical shift effects. It may be difficult to even ensure that these characteristics of the stimuli are present given the numbers of subjects used in most rodent and monkey studies and the difficulty of completely counterbalancing the assignments of different exemplars of each dimension.

Part of the popularity of the ED/ID task is that it provides a means to translate behavioural findings from rodents and nonhuman primates to humans using similar, or in some cases identical, test procedures. This task has also shown a high degree of consistency across species in terms of lesion effects in the frontal cortex (Birrell & Brown, 2000; Brown & Bowman, 2002; Dias, Robbins, & Roberts, 1996). We are not suggesting that the ED/ID task, despite the aforementioned shortcomings, is not useful as a probe of frontal cortex function. However, it may be worth considering whether extradimensional shift deficits after frontal cortical lesions reflect an impairment in flexibility of behavioural strategy, rather than an impairment in shifting attention between perceptual dimensions. This would be congruent with findings in other paradigms attributing strategy switching to the same regions of prefrontal cortex as those implicated in extradimensional shifting (Ragozzino, 2002; Stefani, Groth, & Moghaddam, 2003). For example, set-shifting procedures in rodents use stimuli from at least partially differing sensory modalities, which may also be spatially separated (Barense, Fox, & Baxter, 2002; Birrell & Brown, 2000). When discrimination problems of this type are employed with monkeys and humans, they may use stimuli composed of superimposed images with different qualities (e.g., lines and shapes) to make up the dimensions (Roberts et al., 1988; cf. Rogers et al., 1999). Thus, these types of discrimination procedures may permit different strategies to be used to discriminate different stimulus dimensions—for example, sampling the stimuli in different ways for different dimensions (for rats, whisking with vibrissae vs. sniffing) or directing vision spatially to different parts of the compound stimulus. The question of what is actually represented by performance in an extradimensional shift discrimination is germane to issues of comparative cognition, in which tasks are designed to tap into similar cognitive processes across species (e.g., Olton et al., 1992). Further consideration of the aspects of perceptual attention and behavioural strategy that are engaged by these types of task is warranted, in order to increase their utility for understanding human brain systems, as well as their predictive validity as animal models of human neuropsychiatric conditions.
